# Caveolin-1 deficiency impairs synaptic transmission in hippocampal neurons

**DOI:** 10.1186/s13041-021-00764-z

**Published:** 2021-03-16

**Authors:** Soulmee Koh, Wongyoung Lee, Sang Myun Park, Sung Hyun Kim

**Affiliations:** 1 Department of Neuroscience, Graduate School, Kyung Hee University, Seoul, 02447 South Korea; 2 Department of Pharmacology, Ajou University School of Medicine, Suwon, 16499 South Korea; 3 Department of Physiology, School of Medicine, Kyung Hee University, Seoul, 02447 South Korea; 4 Medical Research Center for Bioreaction To Reactive Oxygen Species and Biomedical Science Institute, School of Medicine, Kyung Hee University, Seoul, 02447 South Korea

**Keywords:** Caveolin-1, Lipid raft, Synaptic vesicle exocytosis, Synaptic vesicle endocytosis, Synaptic transmission

## Abstract

In addition to providing structural support, caveolin-1 (Cav1), a component of lipid rafts, including caveolae, in the plasma membrane, is involved in various cellular mechanisms, including signal transduction. Although pre-synaptic membrane dynamics and trafficking are essential cellular processes during synaptic vesicle exocytosis/synaptic transmission and synaptic vesicle endocytosis/synaptic retrieval, little is known about the involvement of Cav1 in synaptic vesicle dynamics. Here we demonstrate that synaptic vesicle exocytosis is significantly impaired in Cav1–knockdown (Cav1–KD) neurons. Specifically, the size of the synaptic recycled vesicle pool is modestly decreased in Cav1–KD synapses and the kinetics of synaptic vesicle endocytosis are somewhat slowed. Notably, neurons rescued by triple mutants of Cav1 lacking palmitoylation sites mutants show impairments in both synaptic transmission and retrieval. Collectively, our findings implicate Cav1 in activity-driven synaptic vesicle dynamics—both exocytosis and endocytosis—and demonstrate that palmitoylation of Cav1 is important for this activity.

**Supplementary Information:**

The online version contains supplementary material available at 10.1186/s13041-021-00764-z.

## Introduction

Lipid rafts are sphingolipid-cholesterol–enriched subcompartments in the plasma membrane with a size on the scale of several nanometers to micrometers [1]. Biochemically, they are detergent-insoluble complexes that also contain proteins, such as glycosylphosphatidylinositol (GPI)-anchored proteins and transmembrane proteins. Lipid rafts are known to be involved in a range of cellular functions, most notably including membrane trafficking and signaling. Lipid rafts also provide docking sites for membrane fusion [2]. Specifically, components of the membrane-fusion machinery (i.e., VAMP2, SNAP25, syntaxin1A) often localize to this membrane compartment and regulate exocytosis by interacting with lipid rafts [3].

A distinct type of lipid raft is caveolae, which are small (60–80 nm diameter) flask-shaped invaginations in membrane domains [4]. A major component of caveolae is caveolin (Cav), a family of a lipid raft-associated scaffolding proteins comprising Cav1, Cav2 and Cav3, which form homo- and heterodimers in lipid rafts [5]. Cav1 is a 178-amino-acid protein that binds to a fatty acid- and cholesterol-containing microdomain via palmitoyl tails on three cysteine residues, C133, C143, and C156. Cav1 is a major constituent of caveolae, which are present in various cell types, although they are generally less abundant in neurons [6]. Notably, some reports have suggested that Cav1 plays a role in neurons distinct from its function in caveolae [7].

The presynaptic membrane is a highly dynamic area that is important for the proper function of neurons. For example, activity-driven synaptic vesicle fusion and recycling in nerve terminals is an essential process underlying synaptic communication. When an action potential arrives at a nerve terminal, synaptic vesicle membranes fuse to the plasma membrane. Thereafter, the plasma membrane invaginates, reforming synaptic vesicles via an endocytic process. Given the prominence of Cav1 in plasma membrane domains and the essential role of membrane dynamics in nerve terminals, it is reasonable to infer that Cav1 might be involved in this dynamic process.

In this study, we monitored synaptic vesicle dynamics in the absence of Cav1. Neurons lacking Cav1 exhibited impaired synaptic vesicle exocytosis, manifesting as suppressed synaptic transmission and a decreased rate of synaptic vesicle exocytosis. We further found that synaptic vesicle endocytosis is also altered in Cav1-depleted neurons. Finally, we demonstrated that palmitoylation of Cav1 is important for proper synaptic function.

## Methods

### Primary neuron culture

Hippocampal CA1-CA3 regions were isolated from neonatal (0–1 day old) Sprague–Dawley rats (DBL, Strain code: NTac:SD) and plated on poly-ornithine–coated coverslips. Neurons were transfected 8 days after plating and further incubated for 14–21 days in culture medium, as described previously [8]. Neurons were imaged 14–21 days after plating. All results are from at least three independent primary cultures. All animal procedures in this study were performed in accordance with the Guide for the Care and Use of Laboratory Animals with the approval of the Institutional Animal Care and Use Committee of Kyung Hee University.

### Plasmids and transfection

Small hairpin (inhibitory) RNA against Cav1 (shRNA-Cav1) with the targeting sequence, 5′-GAT TGA TCT GGT CAA CCG C-3′ [9], was synthesized, annealed, and ligated into the pSuper vector according to the manufacturer’s instructions. The palmitoylation sites-deleted Cav1 triple mutant, Cav1-C133A/C143A/C156A [10], was constructed by site-directed mutagenesis kit (Stratagene) using mCherry-tagged WT Cav1 (Cav1-mCh) as a template. For optical imaging, primary cultured hippocampal neurons were transfected with the indicated constructs using the Ca^2+^ phosphate precipitation method, as previously described [8]. Briefly, plasmids were incubated with 2X HEBS (273 mM NaCl, 9.5 mM KCl, 1.4 mM Na_2_HPO_4_·7H_2_O, 15 mM D-glucose, 42 mM HEPES pH 7.10) containing 2 mM Ca^2+^, after which the mixture was applied to hippocampal neurons cultured for 8 days in vitro (DIV8). For western blot analysis, primary neurons were transfected by electroporation using a NEPA21 system, as described by the manufacturer (Bulldog Bio).

### Immunofluorescence

For immunofluorescence analyses, DIV14-18 neurons were fixed with 4% paraformaldehyde, permeabilized with 0.2% Triton X-100, and blocked with 5% bovine serum albumen (BSA). After incubating with anti-synapsin1 primary antibodies (BD Bioscience), neurons were incubated with Alexa 488-conjugated secondary antibodies (Invitrogen).

### Lipid raft labeling

Neuronal lipid rafts were labeled by incubating DIV14-18 neurons with the lipid raft marker, GM1, using a Vybrant Alexa Fluor 594 Lipid Raft Labeling Kit (Thermo Fisher) according to the manufacturer’s instructions. Briefly, neurons were incubated for 30 min at 4 °C with Alexa-594–conjugated cholera toxin subunit B (CT-B), which specifically binds to the plasma membrane ganglioside, GM1, an integral part of lipid rafts. Thereafter, cells were incubated with an anti-CT-B antibody for 30 min at 4 °C [11].

### Optical imaging

For immunofluorescence, images of fixed neurons were acquired using a Leica DMRBE microscope with a PL Fluor 40x (1.0 NA.) objective equipped with a CoolSNAP HQ camera (Photometric) driven by MetaMorph software. For pHluorin assays in nerve terminals, live-cell imaging was performed on DIV14-21 neurons transfected with Physin-pH and the indicated plasmids 8 days after plating. Coverslips were mounted in a laminar-flow-perfused stimulation chamber on the stage of a custom-built, laser-illuminated epifluorescence microscope (Zeiss Observer). Live-cell images were acquired with an Andor iXon Ultra 897 (Model #DU-897U-CS0-#BV) back-illuminated EMCCD camera. A diode-pumped OBIS 488 laser (Coherent), shuttered by synchronizing the TTL on/off signal from the EMCCD camera during acquisition, was utilized as a light source. Fluorescence excitation/emission and collection were achieved using a 40 × Fluar Zeiss objective lens (1.3 NA) and 500–550 nm emission and 498 nm dichroic filters (Chroma). Action potentials (APs) were evoked by passing a 1-ms current pulse through platinum-iridium electrodes from an isolated current stimulator (World Precision Instruments). Neurons were perfused with Tyrode’s buffer consisting of 119 mM NaCl, 2.5 mM KCl, 2 mM CaCl_2_, 2 mM MgCl_2_, 25 mM HEPES, 30 mM glucose, 10 μM 6-cyano-7-nitroquinoxaline-2,3-dione (CNQX), and 50 μM D,L-2-amino-5-phosphonovaleric acid (AP5), adjusted to pH 7.4. All experiments were carried out at 30 °C; all images were acquired at 2 Hz with a 50-ms exposure; and all chemicals were purchased from Sigma unless otherwise specified.

### Image analysis

The results of pHluorin-based assays were analyzed as previously described [12], with minor modifications. Images were analyzed using Image J (http://rsb.info.nih.gov/ij) with the Time Series Analyzer plugin, available at https://imagej.nih.gov/ij/plugins/time-series.html. Differences in the expression levels of Cav1 in the presence and absence of shRNA-Cav1 were measured using Image J. Synaptic boutons of neurons were selected as oval regions of interest (diameter, 10 pixels), and the intensity of pHluorin-based fluorescence at synapses was measured and analyzed using Origin Pro 2020. The kinetics of endocytosis and exocytosis were fitted using a single exponential decay function.

### Western blotting

Hippocampal CA3-CA1 regions were dissected from 1-day-old Sprague–Dawley rats, dissociated, plated onto poly-ornithine–coated 6-well dishes, and incubated for 14–18 days. At DIV8, neurons in three wells were transfected with control plasmids and neurons in the other three wells were transfected with shRNA-Cav1. Cells were further incubated until DIV14-18. All experiments were performed in parallel under the same conditions. Cells were lysed with lysis buffer containing 10 mM Tris (pH 7.4), 1% SDS, 10 mM NaF and 1 mM PMSF, supplemented with a protease inhibitor mixture (Complete Mini; Roche, Germany). The protein concentration in lysates was determined using a bicinchoninic acid (BCA) assay (Thermo, IL). Lysate samples containing equal amounts of protein were subjected to sodium dodecyl sulfate–polyacrylamide gel electrophoresis (SDS-PAGE) and subsequently transferred to polyvinylidene difluoride (PVDF) membranes. Membranes were blocked by incubating with 5% nonfat dry milk, and subsequently incubated with anti-Cav1 (BD Biosciences) and anti-β-actin (Santa Cruz, CA) primary antibodies. Band intensities were quantified densitometrically, and the [Cav1]/[β-actin] ratio was measured after background subtraction.

### Statistical analyses

Statistical analyses were performed using either one-way analysis of variance (ANOVA) or Student’s t test, as appropriate. Error bars indicate standard errors of the mean (SEM).

## Results

### Cav1 is localized to nerve terminals in primary cultured hippocampal neurons

**Fig. 1:**
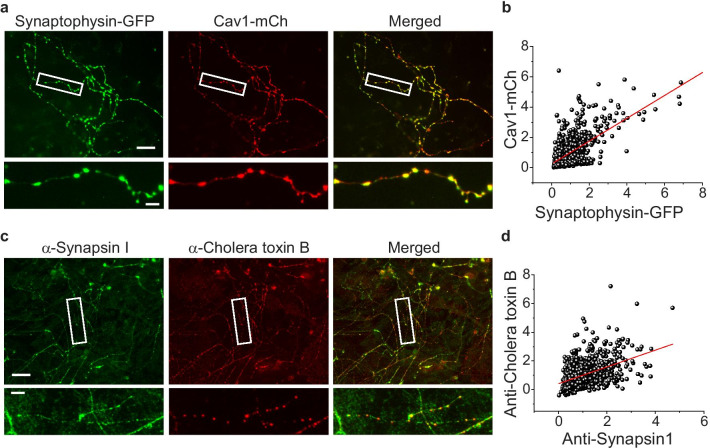
Cav1 co-localizes with lipid rafts at nerve terminals in primary cultured hippocampal neurons. **a** Representative images of exogenous synaptophysin-GFP (left), a nerve terminal marker, together with exogenous Cav1-mCherry (Cav1-mCh; middle) in primary cultured hippocampal neurons. Neurons were co-transfected with synaptophysin-GFP and Cav1-mCh at DIV8 and fixed at DIV14-18. **b** Correlation between synaptophysin-GFP and Cav1-mCh expression levels. **c** Representative images of synapsin I and lipid rafts (CT-B) in primary cultured hippocampal neurons. Neurons were stained with Alexa-Fluor 594–conjugated CT-B (red) and its antibodies, and subsequently immunostained using an anti-synapsin I (green) antibody (SYSY) and Alexa-labeled secondary antibody (Alexa-488). **d** Correlation between synapsin I and CT-B expression levels. Scale bar: 10 μm (up) and 2 μm (bottom)

The function of Cav1 as a component of lipid rafts has not been extensively explored in the nervous system, reflecting the fact that its existence and localization in the membrane of neurons has not been firmly established [6]. Before investigating the physiological role of Cav1 in synapses, we assessed the lipid raft localization of Cav1. First, Cav1-mCh together with the presynaptic marker, synaptophysin-GFP, was introduced into primary cultured hippocampal neurons. After further incubating and then fixing neurons at 14–18 days in vitro (DIV14-18), synaptic co-localization of Cav1 with the presynaptic marker, synaptophysin, was assessed. This assessment showed that Cav1 is highly co-localized with synaptophysin (Fig. 1a), a finding confirmed by correlation analyses (Fig. 1b). We next assessed the distribution of lipid rafts at synapses by incubating neurons with cholera toxin B (CTxB), a known lipid raft-specific marker [13], and labeling synapses using an anti-synapsin I antibody. The distribution of CTxB closely corresponded with that of anti-synapsin1 signals (Fig. 1c, d), suggesting that Cav1 is indeed localized to synapses.

### Cav1 is involved in synaptic vesicle exocytosis at CNS synapses

**Fig. 2:**
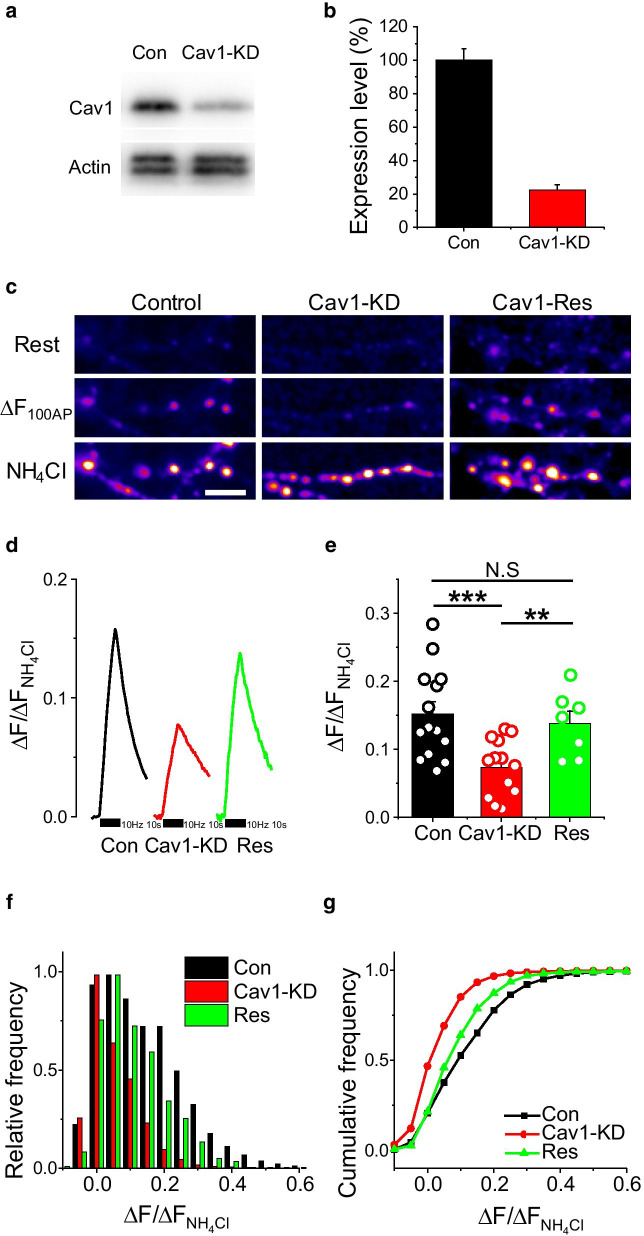
Synaptic vesicle exocytosis is slowed in Cav1-KD neurons. **a** Representative images of western blotting. Neurons transfected with shRNA-Cav1 were lysed, after which proteins in lysates were resolved by SDS-PAGE and transferred to PVDF membranes. Membranes were subsequently incubated with anti-Cav1 antibody and anti-β-actin antibody. **b** Cav1 expression levels in control and Cav1-KD neurons. Intensities of Cav1 bands expressed relative to those of β-actin are presented as means ± SEMs. ([Cav1_Con_] = 100.00 ± 6.8; [Cav1_KD_] = 22.45 ± 3.2, n = 4). **c** Representative images of Physin-pH at synaptic boutons in Control, Cav1-KD, and Cav1-Rescue (Cav1-Res) neurons under basal conditions (top), with stimulation at 10 Hz for 10 s (100 APs; middle), and following application of NH_4_Cl (bottom). Scale bar: 10 μm. **d** Representative trace of Physin-pH responses to 100 APs in Control (black), Cav1-KD (red), and Cav1-Res (green) neurons. **e** Quantification of synaptic transmission, expressed as means ± SEMs in Control (0.15 ± 0.02; n = 14 cells), Cav1-KD (0.07 ± 0.01; n = 14 cells) and Cav1-Res (0.14 ± 0.02; n = 7 cells) neurons (***p < 0.001, **p < 0.01). **f** and **g** Cumulative frequency of exocytosis from single synaptic bouton analyses of Control, Cav1-KD, and Cav1-Res neurons

To investigate the functional role of Cav1 at synapses, we first constructed shRNA targeting Cav1 (shRNA-Cav1) for Cav1 knockdown (KD) in cultured neurons. shRNA-Cav1 was delivered into neurons by electroporation, and the efficacy of Cav1 knockdown was assessed by western blotting (Fig. 2a), which showed that Cav1 expression in Cav1-KD neurons was decreased to ~ 20% of that in parental neurons (Fig. 2b). Next, we monitored synapse function using the Synaptophysin-pHluorin (Physin-pH) system, which has been used effectively to monitor the presynaptic physiological processes of synaptic vesicle exocytosis (synaptic transmission) and synaptic vesicle endocytosis (synaptic retrieval) [14]. First, to verify that Cav1 is involved in synaptic transmission, we co-transfected neurons with Physin-pH and shRNA-Cav1 at DIV8 and 1 week thereafter monitored synaptic transmission in response to stimulation of neurons with 100 action potentials (APs) at 10 Hz. Interestingly, activity-driven synaptic vesicle exocytosis was strongly suppressed in Cav1-KD neurons, which exhibited responses that were ~ 50% of those in controls (Fig. 2c–e). Consistent with this, single synaptic bouton analyses revealed a significant decrease in the distribution of individual bouton responses in Cav1-KD neurons (Fig. 2f, g), implicating Cav1 in the synaptic transmission process.

To determine whether the synaptic transmission defect in Cav1-KD neuron is a direct result of the Cav1 deficiency, we performed rescue experiments in which we assessed synaptic vesicle exocytosis in response to stimulation with 100 APs at 10 Hz in Cav1-KD neurons exogenously expressing shRNA-insensitive Cav1. These experiments clearly showed that exogenous expression of shRNA-insensitive Cav1 restored synaptic transmission to approximately control levels in neurons lacking endogenous Cav1 (Fig. 2c–g and Additional file 1: Fig. S1), demonstrating that Cav1 is indeed involved in synaptic vesicle exocytosis in hippocampal neurons.

### Synaptic vesicle exocytosis rate is decreased in Cav1-KD neurons

Because synaptic vesicle release is suppressed in Cav1-KD neurons, we next tested whether the kinetics of synaptic vesicle exocytosis, which is a continuous synaptic transmission process, is impacted in Cav1-KD neurons. Neurons transfected with Physin-pH with or without shRNA-Cav1 were subjected to prolonged stimulation in the presence of the endocytosis inhibitor bafilomycin, which allowed us to monitor the exocytosis process in isolation [15]. As expected, synaptic vesicle exocytosis was significantly slowed (~ 1.8 fold) in Cav1-KD neurons compared with control neurons. In addition, the functional synaptic pool of recycling vesicles was also slightly decreased in Cav1-KD neurons. These results suggest that Cav1 is also involved in the synaptic vesicle release process and synaptic vesicle pool function.

### Synaptic vesicle endocytosis rate is slowed in Cav1-KD neurons

**Fig. 3:**
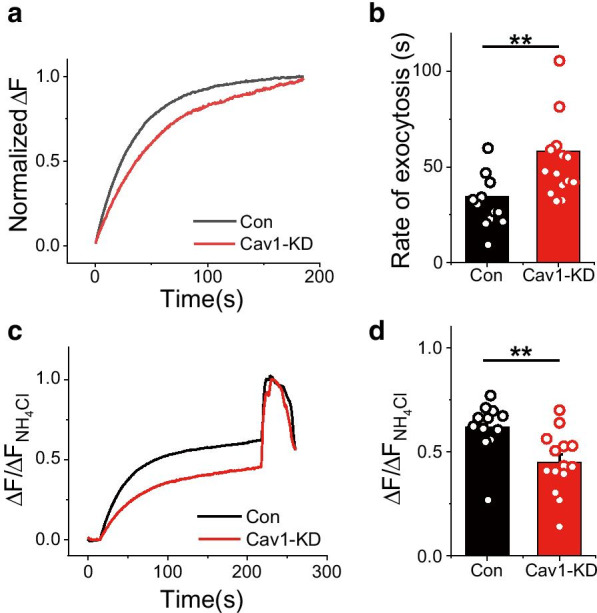
Synaptic vesicle exocytosis rate is significantly decreased in Cav1-KD neurons. **a** Representative normalized traces of Physin-pH exocytosis from Control (black) and Cav1-KD (red) neurons. **b** Quantification of exocytosis, expressed as mean values of time constants (τ) ± SEM, in Control (τ_exo_, 34.61 ± 4.23; n = 12 cells) and Cav1-KD (τ_exo_, 58.12 ± 5.84; n = 14 cells) neurons. **c** Representative traces of Physin-pH responses to 2000 APs at 10 Hz in the presence of bafilomycin A1 (BAF) in control and Cav1-KD neurons. **d** Quantification of recycling vesicle pool size expressed as means ± SEMs in Control (0.62 ± 0.04; n = 12 cells) and Cav1-KD (0.45 ± 0.04; n = 14 cells) neurons (**p < 0.01)

**Fig. 4:**
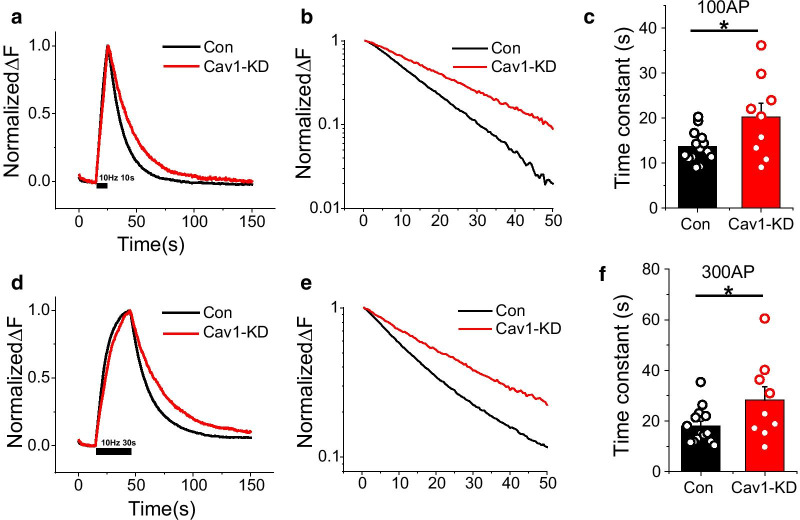
Synaptic vesicle endocytosis kinetics are slowed in Cav1-KD neurons. **a**, **d** Representative traces of Physin-pH responses to 100 APs (**a**) and 300 APs (d) in Control and Cav1-KD neurons. Neurons transfected with Physin-pH with or without shRNA-Cav1 were stimulated with 100 or 300 APs at 10 Hz. **b**, **e** The traces of Physin-pH responses to 100 APs (**b**) and 300 APs (**e**) were projected to the semi-log scale for clearance. (C, D) Quantification of post-stimulus endocytic time constants, expressed as means ± SEMs in (**c**) Control (τ_endo_, 13.71 ± 0.92; n = 14 cells) and Cav1-KD (τ_endo_, 20.29 ± 2.99; n = 9 cells) neurons stimulated with 100 APs, and (**d**) Control (τ_endo_, 18.04 ± 1.90; n = 14 cells) and Cav1-KD (τ_endo_, 28.21 ± 5.28; n = 9 cells) neurons stimulated with 300 APs (*p < 0.05)

Next, we determined whether Cav1 is also involved in the synaptic vesicle endocytosis process. To demonstrate the role of Cav1 in synaptic retrieval, we again applied a pHluorin-based assay. In these experiments, Cav1-KD neurons co-expressing Physin-pH were stimulated with 100 or 300 APs at 10 Hz and then the synaptic vesicle endocytic process was assessed by monitoring the decay of Physin-pH fluorescence and fitting the results to a single exponential decay function. This analysis showed that a Cav1 deficiency in neurons slowed the kinetics of synaptic vesicle endocytosis following AP stimuli (100 or 300 APs) (Fig. 3), decreasing the endocytosis rate by ~ 1.5-fold compared with control neurons (Fig. 4).

### Palmitoylation of Cav1 is involved in Cav1 modulation of synaptic function

**Fig. 5:**
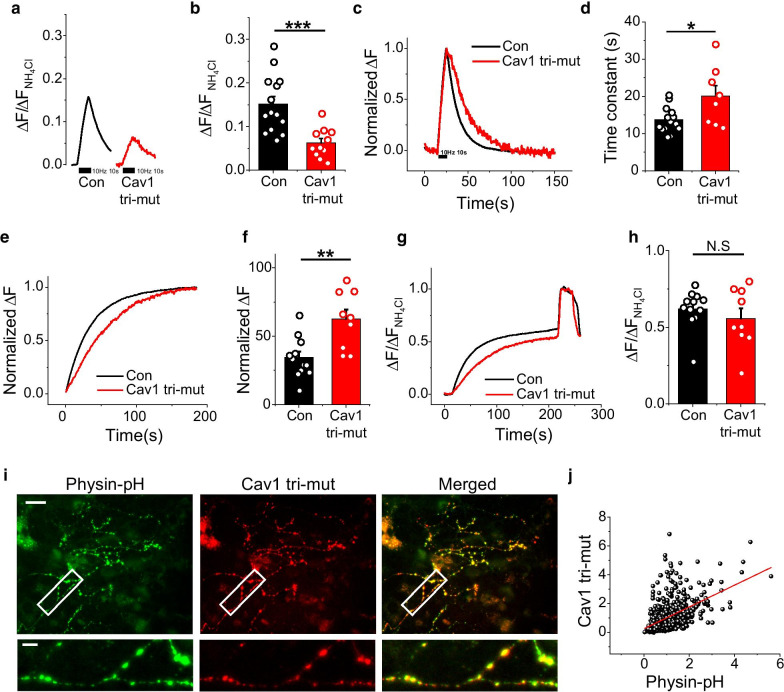
Expression of the Cav1 palmitoylation mutant, Cav1 tri-mut, fails to rescue synaptic transmission and retrieval defects in neurons. **a** Representative traces of exocytosis from Control (black) and Cav1 tri-mut (red)–expressing neurons. Neurons were transfected with Physin-pH with or without shRNA-Cav1 and Cav1 tri-mut and stimulated with 100 APs at 10 Hz. **b** Quantification of synaptic transmission expressed as means ± SEMs in Control (black; 0.15 ± 0.02; n = 14 cells) and Cav1 tri-mut (red; 0.06 ± 0.01; n = 11 cells) neurons. **c** Representative traces of Physin-pH responses to 100 APs in Control (black) and Cav1 tri-mut (red) neurons. **d** Quantification of post-stimulus endocytic time constants, expressed as means ± SEMs in Control (τ_endo_, 13.71 ± 0.92; n = 14 cells) and Cav1 tri-mut (τ_endo_, 20.06 ± 2.79; n = 8 cells) neurons. **e** Representative normalized traces of Physin-pH exocytosis in Control (black) and Cav1 tri-mut (red) neurons. **f** Quantification of time constants of exocytosis expressed as means ± SEMs in Control (τ_exo_ = 34.61 ± 4.23; n = 12 cells) and Cav1-KD (τ_exo_ = 62.56 ± 7.01; n = 9 cells) neurons. **g** Representative traces of Physin-pH responses to 2000 APs at 10 Hz in the presence of bafilomycin A1 (BAF) in control and Cav1 tri-mut neurons. **h** Quantification of recycling vesicle pool sizes expressed as means ± SEMs. in Control (0.62 ± 0.04; n = 12 cells) and Cav1 tri-mut (0.56 ± 0.07; n = 9 cells) neurons. **i** Representative images of primary cultured hippocampal neurons exogenously co-expressing Cav1 tri-mut and synaptophysin-pH. **j** Correlation between Cav1 tri-mut and synaptophysin-pH expression levels (***p < 0.001, **p < 0.01, *p < 0.05). Scale bar: 10 μm (up) and 2 μm (bottom)

Palmitoylation—the covalent attachment of palmitic acid to cysteine—is among the protein modifications that mediate membrane association [16]. Cav1 has three cysteine sites, C133, C143 and C156, for palmitoylation, which is critical for attachment of Cav1 to the membrane. With this in mind, we examined whether palmitoylation of Cav1 is necessary for Cav1 modulation of synaptic function. To this end, we mutated the three palmitoylation sites, C133, C143 and C156, in Cav1 to alanine, yielding the non-palmitoylatable triple-mutant (tri-mut), Cav1-C133A/C143A/C156A. To determine whether palmitoylation of Cav1 influences functional synaptic physiology, we replaced endogenous Cav1 with the Cav1 tri-mut by co-transfecting neurons with shRNA-Cav1, Cav1 tri-mut and Physin-pH (Fig. 5i, j and Additional file 1: Fig. S1). We then monitored synaptic vesicle exocytosis and endocytosis in response to stimulation with 100 APs at 10 Hz. As shown in Fig. 5a, b, synaptic vesicle exocytosis in Cav1 tri-mut–expressing neurons was decreased to ~ 6% of that in control neurons expressing endogenous WT Cav1 only. Furthermore, the rate of synaptic vesicle fusion was significantly increased (~ twofold) compared with control neurons (Fig. 5e, f), although the recycling pool size of synaptic vesicles was not changed (Fig. 5g, h), suggesting that palmitoylation-dependent membrane localization of Cav1 is important for synaptic transmission. In addition, the rate of synaptic vesicle endocytosis in Cav1 tri-mut–expressing neurons was also increased ~ 1.5 fold compared with the endocytosis rate in controls (Fig. 5c, d). Collectively, these results indicate that palmitoylation of Cav1 has a critical role in synaptic physiology, impacting both synaptic vesicle exocytosis and endocytosis. Interestingly, despite the absence of palmitoylation, the Cav1 tri-mut was still distributed to the presynaptic area (Fig. 5i, j).

## Discussion

Lipid rafts are specific compartments of the plasma membrane that are known to be involved in membrane trafficking and cellular singling [1]. Cav1 is a key protein in lipid rafts, including caveolae, acting as a scaffolding protein to provide structural support. Cav1 is expressed in various cell types, where its involvement in a number of cell processes, including cellular trafficking and signaling, have been studied [17]; however, Cav1 has been less thoroughly investigated in the central nervous system (CNS).

In the present study, we demonstrated that Cav1 is involved in presynaptic processes. We found that synaptic transmission was significantly suppressed in Cav1-KD neurons, generated by introducing shRNA targeting Cav1, as evidenced by approximately a 50% decrease in synaptic vesicle exocytosis compared with control neurons. This defect in synaptic vesicle exocytosis was rescued by re-introducing an shRNA-insensitive form of Cav1 into Cav1-KD neurons. In addition, the speed of synaptic vesicle fusion was also impaired in Cav1-KD neurons. We further assessed the synaptic vesicle endocytosis process, showing that the kinetics of synaptic vesicle endocytosis was modestly slowed in Cav1-KD neurons compared with control neurons. Similar alterations of synaptic vesicle trafficking were observed in Cav1-KD cells expressing the Cav1 tri-mut, lacking three palmitoylatable cysteines, indicating that palmitoylation is essential for normal Cav1 function. Collectively, these findings implicate palmitoylated Cav1 in synaptic vesicle trafficking at CNS synapses (Additional file 2: Fig. S2 and Additional file 3: Fig. S3).

Although this study has uncovered some important functions of Cav1 in presynaptic terminals, several questions remain to be addressed. First, how does Cav1 affect synaptic transmission? The expectation is that this may depend on the membrane composition of the release site. It has been reported that lipid rafts regulate exocytosis through spatial regulation of SNARE complexes, which are responsible for vesicle fusion [1]. This would suggest that Cav1 might control lipid raft formation and distribution of SNARE complexes in lipid rafts. Consistent with this, Cav1 function can also be linked to the synaptic vesicle endocytosis process. It is also possible that Cav1 controls the lipid content of synaptic vesicles, a supposition that would require a careful analysis of the lipid components of synaptic vesicles. Alternatively, Cav1 may associate with synaptic vesicle proteins. The next question arising is how does Cav1 dynamically regulate lipid rafts at synapses and is this regulation related to neural activity? In this context, Cav1-dependent lipid rafts of various sizes are incorporated into synaptic vesicles during endocytosis, and Cav1 is able to interact with endocytic components, which modulates synaptic vesicle endocytosis. A further question is whether the synaptic function of Cav1 related to a neural disorder. Studies have revealed that Cav1 or lipid rafts are implicated in neurodegenerative diseases such as Alzheimer’s disease (AD) and Parkinson’s disease (PD) [18–20], and that Cav1 is also involved in non-neural diseases such as cancer and diabetes [21, 22]. The remaining questions are challenging, but emerging high-resolution imaging (e.g. super-resolution microscopy) technology might provide an approach for addressing them.

## Supplementary Information

**Additional file 1: Fig S1.** shRNA-insensitive Cav1 was successfully expressed in Cav1-KD neurons.

**Additional file 2: Fig S2.** Raw data for western blot.

**Additional file 3: Fig S3.** Raw data for other numeric data.

## Data Availability

Please contact author for data requests.

## Abbreviations

CT-B: Cholera toxin subunit B
Physin-pH: Synaptophysin-pHluorin
Cav1: Caveolin-1
BAF: Bafiliomycin1
KD: Knock-down
