# Supplementary material for: Caveolin-1 deficiency impairs synaptic transmission in hippocampal neurons
Source: Mol Brain. 2021 Mar 16;14:53. doi: 10.1186/s13041-021-00764-z (PMC7962241; doi:10.1186/s13041-021-00764-z)
Supplement: Supplementary file 1 — Additional file 1: Fig S1. shRNA-insensitive Cav1 was successfully expressed in Cav1-KD neurons. [file 13041_2021_764_MOESM1_ESM.docx]

**Fig S1. shRNA-insensitive Cav1 was successfully expressed in Cav1-KD neurons.**

(A and B) Representative images of rescued neurons. Neurons expressing Cav1-KD, shRNA-insensitive Cav1-Res-mCh (A), or Cav1 tri-mut-mCh (B) and Physin-pH were fixed and imaged. (First panel) Physin-pH image of the resting condition in rescued neurons. (Second panel) Physin-pH image of responsive synaptic boutons at 100AP in rescued neurons. (Third panel) Physin-pH image in the presence of NH_4_Cl for visualizing entire synaptic boutons in rescued neurons. (Fourth panel) corresponding images of Cav1-mCh in the same rescued neurons. Scale bars represent 20 µm (up) and 2 µm (down).
