# Supplementary material for: Caveolin-1 deficiency impairs synaptic transmission in hippocampal neurons
Source: Mol Brain. 2021 Mar 16;14:53. doi: 10.1186/s13041-021-00764-z (PMC7962241; doi:10.1186/s13041-021-00764-z)
Supplement: Supplementary file 2 — Additional file 2: Fig S2. Raw data for western blot. [file 13041_2021_764_MOESM2_ESM.pdf]

Cav-1

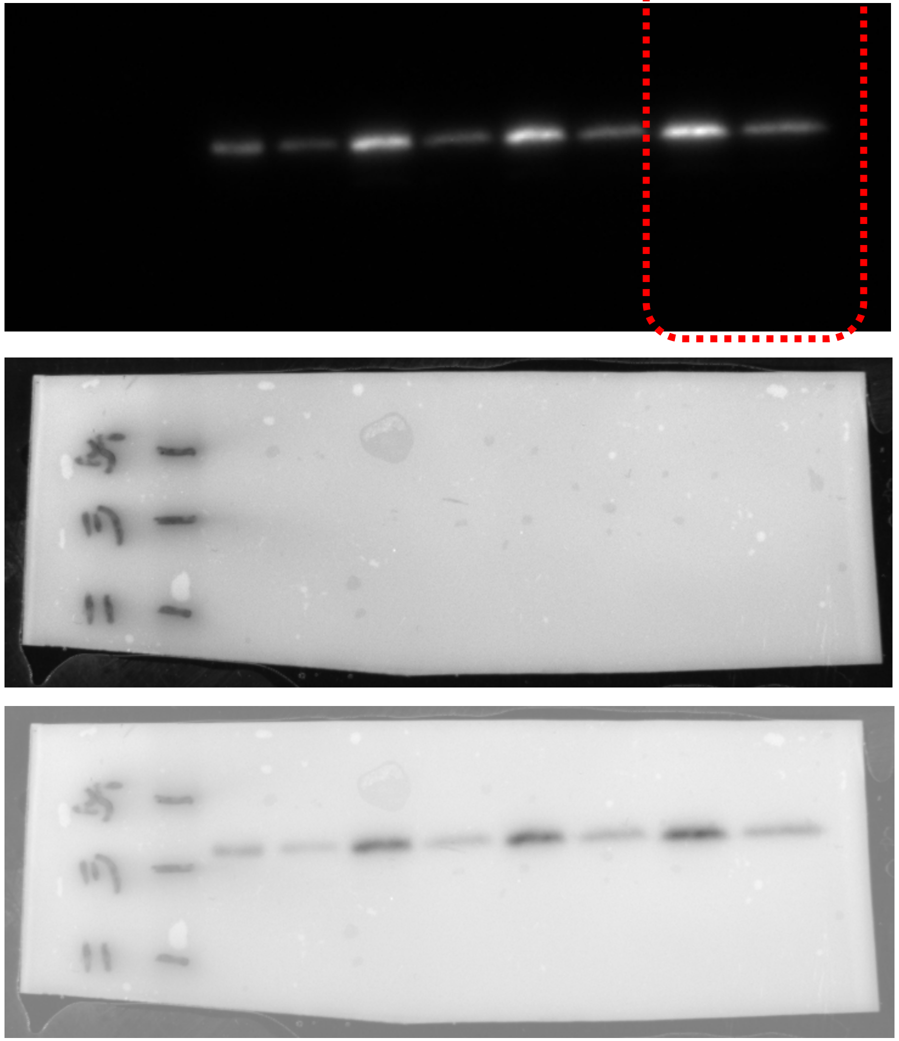
For Figure
Dish No. :
Dish1
Dish2
Dish3
Dish4
kDa
Con KD
Con KD
Con KD
Con KD
chemiluminasecence
PVDF membrane
Overlay

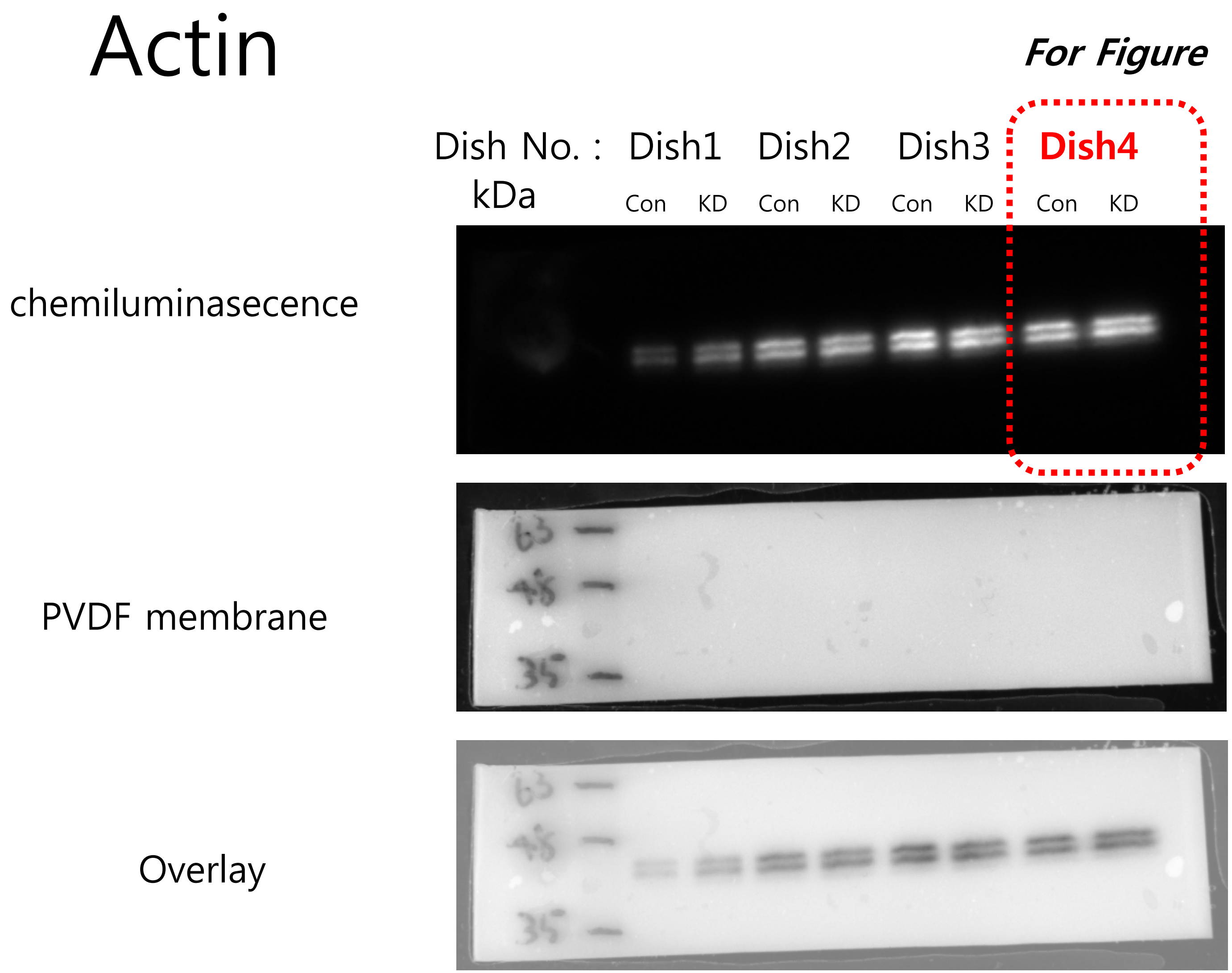
Actin
For Figure
Dish No. :
kDa
Dish1
Dish2
Dish3
Dish4
Con
KD
Con
KD
Con
KD
Con
KD
chemiluminasecence
PVDF membrane
Overlay
63
48
35
